# Oral Immunization with OspC Does Not Prevent Tick-Borne *Borrelia burgdorferi* Infection

**DOI:** 10.1371/journal.pone.0151850

**Published:** 2016-03-18

**Authors:** Rita Melo, Luciana Richer, Daniel L. Johnson, Maria Gomes-Solecki

**Affiliations:** 1 Department of Microbiology, Immunology and Biochemistry, College of Medicine, University of Tennessee Health Sciences Center, Memphis, Tennessee, United States of America; 2 Immuno Technologies Inc., Memphis, Tennessee, United States of America; 3 Molecular Resource Center, University of Tennessee Health Sciences Center, Memphis, Tennessee, United States of America; University of Kentucky College of Medicine, UNITED STATES

## Abstract

Oral vaccination strategies are of interest to prevent transmission of Lyme disease as they can be used to deliver vaccines to humans, pets, and to natural wildlife reservoir hosts of *Borrelia burgdorferi*. We developed a number of oral vaccines based in *E*. *coli* expressing recombinant OspC type K, OspB, BBK32 from *B*. *burgdorferi*, and Salp25, Salp15 from *Ixodes scapularis*. Of the five immunogenic candidates only OspC induced significant levels of antigen-specific IgG and IgA when administered to mice via the oral route. Antibodies to OspC did not prevent dissemination of *B*. *burgdorferi* as determined by the presence of spirochetes in ear, heart and bladder tissues four weeks after challenge. Next generation sequencing of genomic DNA from ticks identified multiple phyletic types of *B*. *burgdorferi* OspC (A, D, E, F, I, J, K, M, Q, T, X) in nymphs that engorged on vaccinated mice. PCR amplification of OspC types A and K from flat and engorged nymphal ticks, and from heart and bladder tissues collected after challenge confirmed sequencing analysis. Quantification of spirochete growth in a borreliacidal assay shows that both types of spirochetes (A and K) survived in the presence of OspC-K specific serum whereas the spirochetes were killed by OspA specific serum. We show that oral vaccination of C3H-HeN mice with OspC-K induced significant levels of antigen-specific IgG. However, these serologic antibodies did not protect mice from infection with *B*. *burgdorferi* expressing homologous or heterologous types of OspC after tick challenge.

## Introduction

Lyme disease is the most reported human vector-borne illness in the US with the current number of probable cases estimated at ~300,000 per year [1]. There is no vaccine available for the prevention of this illness. Several attempts have been made to develop vaccines against Lyme disease that either vaccinate the human host [2], [3], [4] or are intended to disrupt the transmission cycle of *Borrelia burgdorferi* by vaccinating reservoir hosts [5], [6], [7] or impair behavior of the *Ixodes scapularis* tick vector [8], [9]. Oral immunization strategies are the mainstay for vaccination against human viral infections [10] and for development of reservoir targeted vaccines (RTV) [11], [12]. Further, there are examples of effective oral vaccines against bacterial pathogens [13], [14], [15], [16].

Outer surface protein C (OspC) is currently considered one of the most promising Lyme disease vaccine candidates due to the protein being upregulated at 34–37°C rather than 24°C [17], [18]. Furthermore, OspC is necessary to initiate mammalian infection [19], [20], [21] but it is turned off before 28 days post-infection [19]. The OspC role as an inducer of bactericidal antibodies differs between infected humans and mice [22], [23], [24].

In addition to an invasive phyletic type of OspC (type K) we selected OspB and BBK32 from *B*. *burgdorferi* for mammal (human or reservoir) targeted vaccines based on evidence that these proteins induced significant IgG immune responses when administered via a parenteral route [25], [26], [27]. To evaluate candidates for vector-targeted vaccines (VTV), or for combinations of RTV and VTV, Salp25 and Salp15 from *Ix*. *scapularis* were investigated based on observations that Sapl25 is expressed in the tick when it is fully engorged [28] and that Salp15 facilitates infection of the mammalian host [29].

We expanded on technology previously used in our laboratory to design oral vaccines against Lyme disease, leptospirosis and plague [5], [13], [14], [16] and engineered five oral vaccine candidates (OspC, OspB, BBK32, Salp25 and Salp15) in an auxotrophic strain of *E*. *coli*. We analyzed serological and mucosal immune responses to all immunogens and evaluated *B*. *burgdorferi* dissemination in mice after oral immunization with *E*. *coli* delivered OspC type K. Vaccine efficacy was determined after challenge of vaccinated mice with *Ix*. *scapularis* infected with strains of *B*. *burgdorferi* expressing multiple types of OspC.

## Materials and Methods

### Animals and ethics statement

Six to eight week old female C3H-HeN mice (Charles River, Boston) were used. This study was carried out in strict accordance with the recommendations in the Guide for the Care and Use of Laboratory Animals of the National Institutes of Health. The protocol was approved by the University of Tennessee Health Science Center Institutional Animal Care and Use Committee, Animal Care Protocol Application (Permit Number: 14–007). Survival anesthesia was performed under isofluorane or avertin, and all efforts were made to minimize animal suffering. Avertin was used before tick challenge. For euthanasia we used isofluorane followed by a physical method (cervical dislocation, thorachotomy and collection of vital organs). During immunization animals were kept in groups of 4 in standard cages under regular feeding and light cycle schedules. For tick challenge mice were individually caged in wire bottom cages, which were held in FIC-2 isolators for a week. During that week mice were closely monitored for any signs of discomfort for a minimum of three times daily, engorged ticks were collected and precise records were kept for each animal. We maintain a protocol in which animals that become severely ill prior to experimental endpoint (ruffled coat, loss of mobility) are to be humanely euthanized. No animals died prior to the experimental endpoint.

### Culture of *Borrelia burgdorferi*

A culture containing multiple OspC types of *B*. *burgdorferi* originally isolated from heart of *Peromyscus leucopus* infected with field caught ticks (from New York State) was used to produce our infected flat nymph tick cohort. *B*. *burgdorferi* strain BL204 (OspC type K) (kindly provided by Dr. Ira Schwartz, NYMC) and *B*. *burgdorferi* strain B31MI (OspC type A, passage 4) were used in the neutralization assay. *B*. *burgdorferi* were cultured in Barbour-Stoenner-Kelly (BSK-H) medium at 34°C until the cells reached the log phase. The number of spirochetes was determined using dark field microscopy and quantitative PCR.

### Plasmid construction

The genes *ospC* type K (*ospC-K*), *ospB* and *bbk32* from *B*. *burgdorferi* sensu stricto and *salp25* and *salp15* from *Ix*. *scapularis* were synthesized fused to the nucleotide sequence encoding the leader peptide of outer surface protein A (OspA) from *B*. *burgdorferi* and cloned into pET9c (for vaccine production) and into pET28a (for protein purification) using codons optimized for expression in *E*. *coli* (Blue Heron Biotechnology, Inc., WA, USA). DNA constructs were then transformed into the *E*. *coli* strain BL21 (DE3) pLysS. The parental *E*. *coli* strain transformed with the empty plasmid was used as a control (Ctrl).

### Protein purification

Recombinant *E*. *coli* clones were grown in Tryptone Broth Yeast (TBY) medium supplemented with 50 μg/ml Kanamycin (Kn) at 37°C, shaking at 225 rpm, until it reached an OD_600_ of 0.8. The expression of 6xHis—recombinant proteins was induced by adding 1 mM IPTG (isopropyl-β-d-thiogalactopyranoside) to the cells followed by incubation at 37°C for 3h. The cells were harvested by centrifugation at 4000 x g for 10 min at 4°C. The proteins were purified using the Ni-NTA Purification System (Invitrogen) following the manufacturers instructions. Protein concentration was determined by the Bradford protein assay (Bio-Rad, Hercules, CA, USA), and was stored at -80°C.

### Cell fractionation

Recombinant *E*. *coli* were cultivated in TBY medium supplemented with 50 μg/ml Kanamycin at 37°C, 225 rpm. At an OD_600_ = 0.8, cells were induced with 1mM IPTG for 3 hours and grown to an OD_600_ of ~1. The cells were harvested by centrifugation at 20,000g for 10 min at 4°C and were washed three times with ice-cold phosphate buffered salt solution (PBSM, Gibco, Grand Island, NY). The pellet was resuspended in ice-cold PBS supplemented with protease inhibitor cocktail (Complete EDTA-free, Roche Diagnostics GmBH, Germany) to an OD_600_ ~ 1. *E*. *coli* cells were disrupted with a French press (Thermo Electron Corporation, Milford, MA) and centrifuged at 20,000 x g for 10 minutes at 4°C to isolate the cytosol fraction (supernatant) from the cell envelope (pellet). The pellet was resuspended in 1ml of ice-cold PBS 2% Triton-X114 (Sigma Aldrich, St. Louis, MO) (v/v) and was incubated at 0°C for 1 hour with frequent gentle agitation. Phase separation was performed by warming the suspension for 30 minutes in a 37°C water-bath, followed by centrifugation at 13 000 x g for 15 minutes at 25°C. Aqueous phase and detergent phase were then collected, separated from each other and were washed three times. Briefly, the aqueous phase was washed by adding fresh 10% Triton X-114 to a final concentration of 2%. The aqueous phase was rewarmed for 30 minutes in a 37°C water-bath and centrifuged at 13 000 x g for 15 minutes at 25°C. The detergent phase was washed by diluting it to 1 ml in ice-cold PBS followed by rewarming for 30 minutes in a 37°C water-bath and a centrifugation at 13 000 x g for 15 minutes at 25°C. Total extract (TE), Supernatant (SN), Cell envelope (CE), Cell Envelope/Detergent phase (CE/DET), Cell Envelope/Aqueous phase (CE/AQ), and pure recombinant proteins were analyzed on a 10% denaturing polyacrylamide gel and electrotransferred to a polyvinylidene difluoride membrane (PVDF, Millipore, Billerica, MA) for analysis with antigen-specific- polyclonal mouse antibody. Protein was quantified by densitometry using Alpha Imager (*Alpha Innotech*, San Leandro, CA).

### Preparation of oral vaccine and intragastric inoculation

Recombinant *E*. *coli* was cultured in TBY supplemented with Kn, and grown at 37°C, 225 rpm, to an OD_600_ of 0.8. Protein expression was induced with 1 mM IPTG during 3h. The cells were harvested by centrifugation at 4000xg for 10 min at 4°C and resuspended in 20% glycerol/phosphate buffered salt solution (Gibco, Grand Island, NY) in 1% of the initial volume. Cell suspensions in aliquots of 2 ml were frozen quickly in a dry ice bath and stored at -80°C. Aliquots of recombinant *E*. *coli* were thawed at 4°C and 400 μl (10^9^ cells) were placed in a ball-tipped syringe for oral gavage inoculation. Groups of female C3H/HeN mice were immunized by intragastric inoculation of induced recombinant *E*. *coli* or with the empty vector (Ec) as controls. Mice received the first immunization, twice daily, for 10 days (days 1–5 and 8–12), rested for two weeks, received the first boost for 5 days (days 29–33), rested for another week and then received a second boost for 5 days (days 43–47). On day 73, mice were terminated for collection of bronchoalveolar lavage (BAL) or challenged. Low amounts of mouse blood (up to 50 μl) were obtained via tail nick on the following days: 0, 28, 42, 73, 105, 135 and 170 after immunization. Stool (GUT, gastrointestinal tract secretions), bronchoalveolar lavage (BAL) and vaginal lavage (VAL) were collected on day 73 after immunization.

### Production of laboratory infected *Ixodes scapularis*

Media (50 μl) containing 10,000 *B*. *burgdorferi* sensu stricto (previously cultured from heart of *P*. *leucopus* infected with field caught ticks) was injected subcutaneously in the back of the neck of C3H-HeN mice; four weeks later, batches of clean flat larvae (purchased from Oklahoma State University or from the University of Rhode Island) were placed in the back of the head of anesthetized mice; mice were checked to ensure ticks were attached and were caged individually and monitored for one week. Engorged larval ticks (infected at a rate ~83%, confirmed by PCR) were collected as they naturally fell off and allowed to molt to the next stage for about 3 months to produce nymphal ticks.

### Tick challenge

Tick challenge was performed by placing six *B*. *burgdorferi* infected nymphal ticks on the back of the head of anesthetized mice. Five to seven days later, ticks that engorged after taking a blood meal were retrieved after naturally falling off, immediately processed for DNA extraction and stored at -20°C for further q-PCR and direct PCR analysis.

### Serological and mucosal immune responses

Serum, bronchoalveolar lavage (BAL), stool (GUT, gastrointestinal tract secretions) and vaginal lavage (VAL) from mice orally immunized with *E*. *coli* expressing recombinant proteins were tested by indirect ELISA for the presence of IgG and IgA. IgG was further isotyped for IgG1 and IgG2a. Purified recombinant 6xHis-tagged proteins were coated at 2 μg/ml on Nunc MaxiSorp™ flat-bottom ELISA plates (eBioscience, San Diego, CA, USA) and indirect ELISA was performed using serum (1:100), BAL (1:1), VAL (1:1) and GUT (1:1). Goat anti-mouse IgG (1:50,000), goat anti-mouse IgG1 (1:50,000), goat anti-mouse IgG2a (1:50,000) and goat anti-mouse IgA (1:50,000) horseradish peroxidase-conjugated antibody (SouthernBiotech, Birmingham, Alabama) was used as secondary antibody.

### Assessment of *B*. *burgdorferi* dissemination

#### Western blot

Borrelia B31 ViraStripe IgG (OspC type A, Viralab Inc, Oceanside, CA) was performed according to the manufacturer’s instructions using blood from immunized mice as the first antibody. A pattern of 5 out of 10 bands was considered evidence of infection.

#### Culture of *B*. *burgdorferi* from tissues and dark field microscopy

Heart, bladder and ear biopsy tissues were individually cultured in BSK-H medium with an antibiotic mixture for Borrelia (Sigma) for up to 6 weeks at 34°C. Cultures were checked every week by dark field microscopy (AxioImager, Zeiss, Germany). Cultures were deemed positive if Borrelia cells were observed in any field and negative if no Borrelia cells were observed in 10 fields.

#### Quantitative PCR (qPCR)

DNA from heart (25mg), bladder (25mg), ear tissue (2mm), *B*. *burgdorferi* culture, flat and engorged ticks were extracted with DNeasy Blood and Tissue kit according to the manufacturer instructions (Qiagen, Valencia, CA). The purified DNA was stored at -20°C until use. The concentration of *B*. *burgdorferi* was quantified by a Bio-Rad iQ5 Real-time System (Bio-Rad, Hercules, CA) using the iTaq Universal probes supermix (Bio-Rad). The *flaB* and the *ospC* genes were PCR amplified. The PCR reaction contained 10 μM of each primer, 5 μM of the specific probe and 5 μl of DNA in a total volume of 25 μl. The amplification protocol consisted of 10 min at 95°C, followed by 40 cycles of 95°C for 15s and 60°C for 1 min. Results were expressed as the number of *B*. *burgdorferi* per 1mg (heart and bladder), per 1mm (ear) of tissue or per tick.

### Next generation sequencing

Genomic DNA purified from 10 engorged nymphal ticks that fed on immunized mice was sent for next generation sequencing at Genewiz (South Plainfield, NJ) with the following OspC general primers, OspC-f: GGAGGCACAAATTAATG and OspC-r: GACTTTATTTTTCCAGTTAC. The library was prepared using NEBNext Ultra DNA Library Preparation Kit as per the manufacturer’s instructions. Quality control was performed by Qubit dsDNA Assay and the High Sensitivity Assay on the Agilent 2100 Bioanalyser. Library was then sequenced on Illumina MiSeq 2x300 PE cartridge. BOWTIE2 was used to generate the alignment (http://bowtiebio.sourceforge.net/bowtie2/index.shtml). Cufflinks was used to generate the rpkm (reads per kilobase per million) of each strain from the alignment results. Data was deposited in the NIH Sequence Read Archive (SRA) accession Nr. SRP071080.

### Borreliacidal assay

The borreliacidal assay was adapted from a method previously described [30]. *B*. *burgdorferi* B31MI (OspC type A) and BL204 (OspC type K) were grown at 34°C in BSK medium and cell density adjusted to ~ 10^4^ cells per mL. Serum was harvested from mice immunized orally with *E*. *coli* expressing OspC type K and immunized subcutaneously with purified recombinant OspA (αOspA), rOspC type K (αOspCK) and control (αCtrl). Subcutaneous inoculum was prepared by combing 100 μg of purified protein with alum (1:1). The borreliacidal effect of vaccinated serum, pre-challenge (containing active complement) was tested by adding 16 μl of cells to 8 μl of mouse serum and 16 μl of BSK medium in 0.2 mL PCR tubes and incubating the reaction at 34°C for 7 days for visualization of *B*. *burgdorferi* under a dark field microscope and for quantification of growth by qPCR. Aliquots were collected and frozen on days 0, 3, 5, 7. Two (2) μl of each sample were used to quantify the FlaB copies by quantitative PCR reaction method (StepOne Plus, Applied Biosciences).

### Statistical analysis

Data are represented as mean ± standard deviation. Statistical analyses were performed using unpaired T test with equal standard deviation.

## Results

### Characterization of oral vaccine constructs before challenge

#### Localization and quantification of immunogens in the cell envelope of *E*. *coli*

**Fig 1:**
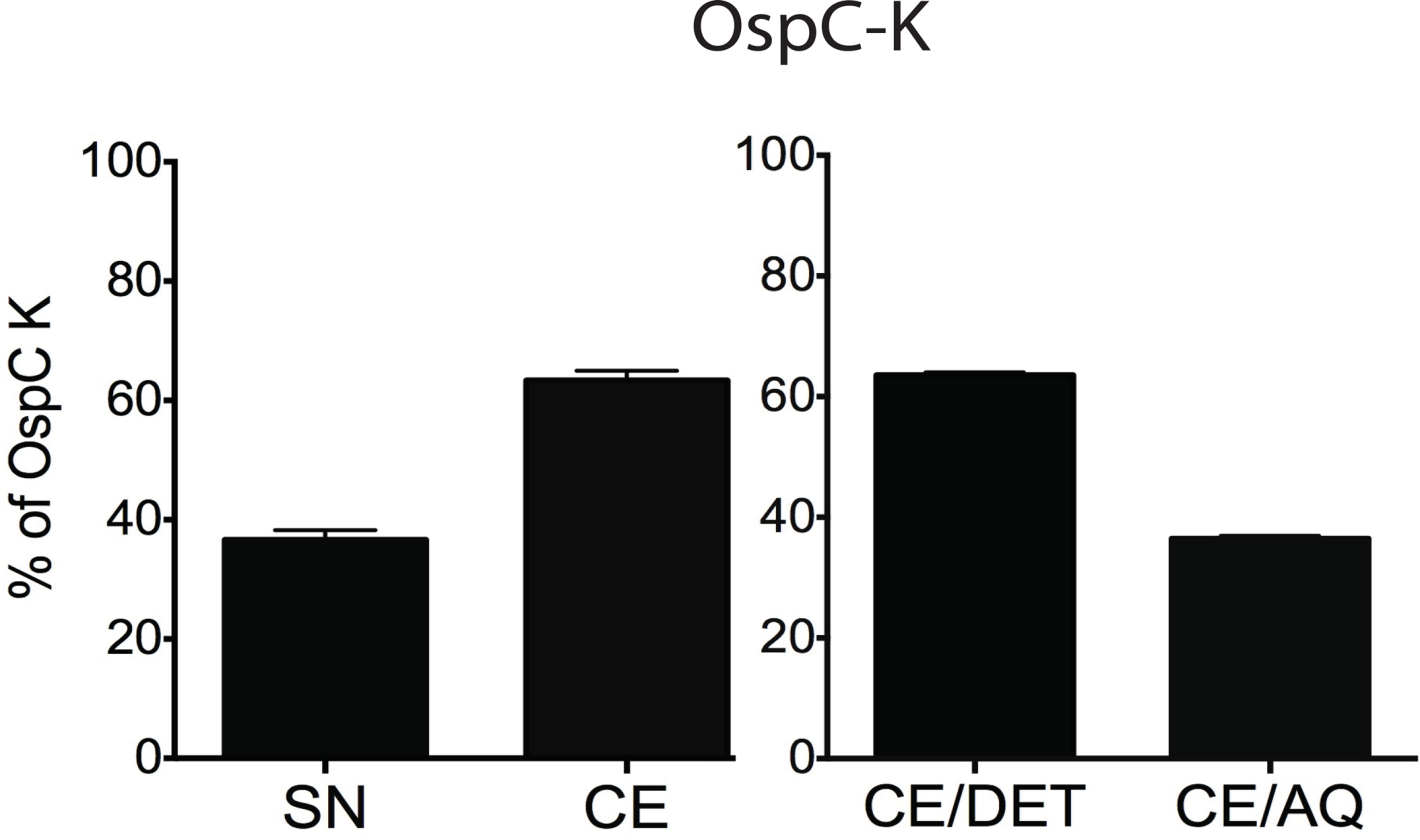
Localization of recombinant OspC in *E*. *coli*. Cells expressing OspC-K were disrupted with a French press. Supernatant (SN), cell envelope (CE), and cell envelope were partitioned into detergent (CE/DET) and aqueous (CE/AQ) phases and protein was quantified by densitometry using an Alpha Imager (Alpha Innotech, San Leandro, CA).

We cloned the outer surface protein C type K (*ospC-K*), the outer surface protein B (*ospB*) and the fibronectin binding protein BBK32 (*bbk32*) genes from *B*. *burgdorferi* sensu stricto into an *E*. *coli* expression plasmid as well as salivary gland proteins 15 and 25 (*salp15* and *salp25*) from *Ix*. *scapularis*. We evaluated the localization of the recombinant proteins within the cell envelope of *E*. *coli* by cell fractionation followed by quantification by densitometry (Fig 1 and S1 Fig). Analysis of cytosol (SN) and cell envelope (CE) fractions revealed that all five recombinant proteins partition primarily in the cell envelope (CE, 60–80%). When the CE was further fractionated by Triton X-114 solubilization and phase partitioning, OspC-K (Fig 1), BBK 32 (S1B Fig) and Salp 15 (S1D Fig) were found primarily in the Triton X-114 hydrophobic, detergent phase of the cell envelope (CE/DET: 76.0%, 61.2% and 80%, respectively), whereas OspB (S1A Fig) and Salp25 (S1C Fig) were found primarily in the aqueous phase of the cell envelope (CE/AQ: 74.3% and 71.7%, respectively).

#### Serological IgG and mucosal IgA antibody response to orally delivered immunogens before challenge

**Fig 2:**
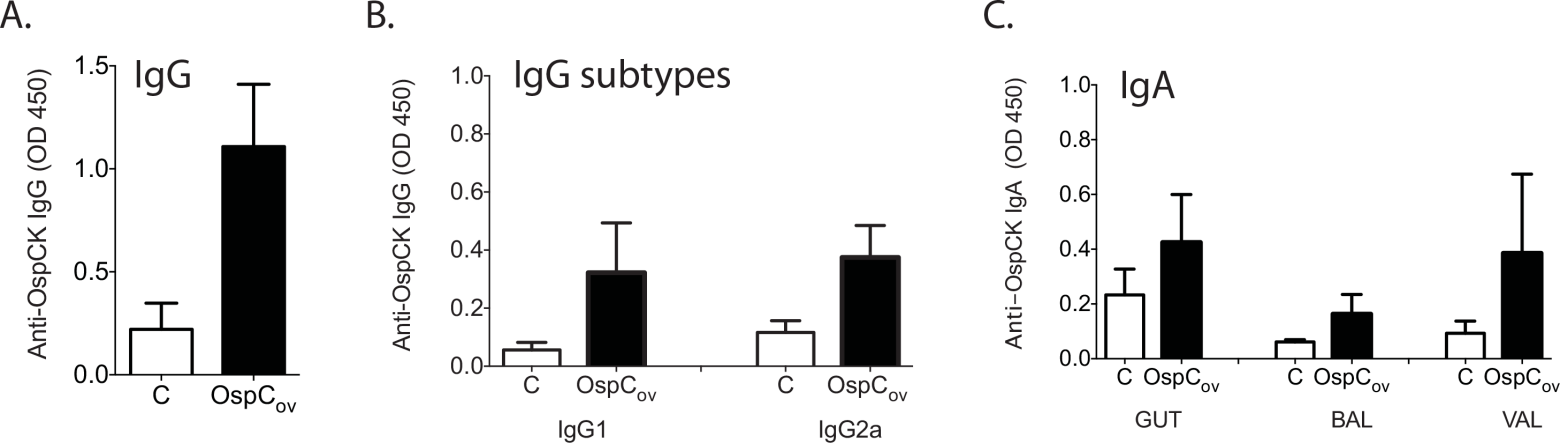
Serological IgG and mucosal IgA response in mice immunized orally with recombinant *E*. *coli* expressing OspC the day before challenge. C3H/HeN mice (n = 12 per group) were vaccinated orally with live recombinant *E*. *coli* expressing *B*. *burgdorferi* gene *ospC* type K (OspC-K) and control mice were orally inoculated with *E*. *coli* carrying the empty vector (C). Blood, stool (GUT), bronchoalveolar lavage (BAL) and vaginal lavage (VAL) samples were collected on the day before challenge and antigen-specific IgG (Fig 2A), subtyped into IgG1 and IgG2a (Fig 2B) and antigen-specific IgA (Fig 2C) were measured by ELISA and plotted as Optical Density at 450 nm (OD 450). The average of triplicate readings per mouse/per group was determined and the error bar indicates standard deviation.

We determined serological IgG and mucosal IgA immune responses induced by oral administration of *E*. *coli* expressing *B*. *burgdorferi* and *Ix scapularis* antigens in C3H-HeN mice as well as in controls that received *E*. *coli* expressing the empty vector on the day before challenge (Fig 2 and S2 Fig). Subcutaneous immunization with purified OspC-K (positive control) had a mean antibody level of OD_450_ = 1.795 (± 0.095 SD). Mice immunized orally with *E*. *coli* expressing OspC-K produced significant levels of OspC-K specific IgG antibodies with a mean antibody level of OD_450_ = 1.108 (± 0.303 SD) (Fig 2A), of which, OD_450_ = 0.322 (± 0.171 SD) was IgG1 and OD_450_ = 0.375 (± 0.110 SD) was IgG2a (Fig 2B). Differences between vaccinated and unvaccinated controls are statistically significant, *p*<0.0001. Oral immunization with *E*. *coli* expressing other *B*. *burgdorferi* genes (*ospB*, *bbk32*) and *Ix scapularis* genes (*salp25*, *salp15*) did not induce levels of antigen-specific IgGs different from the control, *p*>0.05 (S2A Fig) whereas subcutaneous immunization with purified recombinant proteins OspB, BBK32, Salp25 and Salp15 induced significant levels of antigen-specific IgGs (data not shown). We tested levels of antigen-specific IgA in stool (GUT) which measures local IgA response to orally administered antigen, by ELISA. We also measured IgA in bronchoalveolar lavage (BAL) and vaginal lavage (VAL), which measures antibody production distant to the inoculation site. Mice immunized orally with *E*. *coli* expressing OspC-K (Fig 2C) produced significant levels of OspC-K specific IgA antibodies not only locally at the site of immunization (GUT), mean OD_450_ = 0.425 (± 0.175 SD) compared to 0.233 (± 0.094 SD) in the control, *p* = 0.0030; but also at mucosal sites distal to the gut, such as the lung (BAL) with a mean OD_450_ = 0.163 (± 0.071 SD) compared to 0.062 (±0.008 SD) in the control, p = 0.0004, and in the vagina (VAL) with a mean OD_450_ = 0.385 (± 0.289 SD) compared to 0.093 (± 0.044) in the control, *p* = 0.0022. Mice immunized orally with *E*. *coli* expressing *B*. *burgdorferi* genes *ospB* and *bbk32* and *Ix scapularis* genes *salp25* and *salp15* did not produce significant levels of antigen specific IgA (S2B Fig).

### Efficacy of an OspC-based oral vaccine after tick challenge

We determined the efficacy of an *E*. *coli*-based oral vaccine expressing OspC-K in preventing infection with *B*. *burgdorferi* after tick challenge. We used laboratory produced flat nymphs infected with a number of *B*. *burgdorferi*, which had an infection rate of ~83%. We placed 6 flat *Ix scapularis* nymphs per mouse (n = 12, per group), using a total of 144 flat nymphs. After challenge, we recovered 57 of the 72 nymphs placed on the control group and 58 out of the 72 nymphs placed on the immunized group, for an average nymphal recovery rate of 80% and 81%, respectively.

#### Seroconversion to *B*. *burgdorferi*

**Fig 3:**
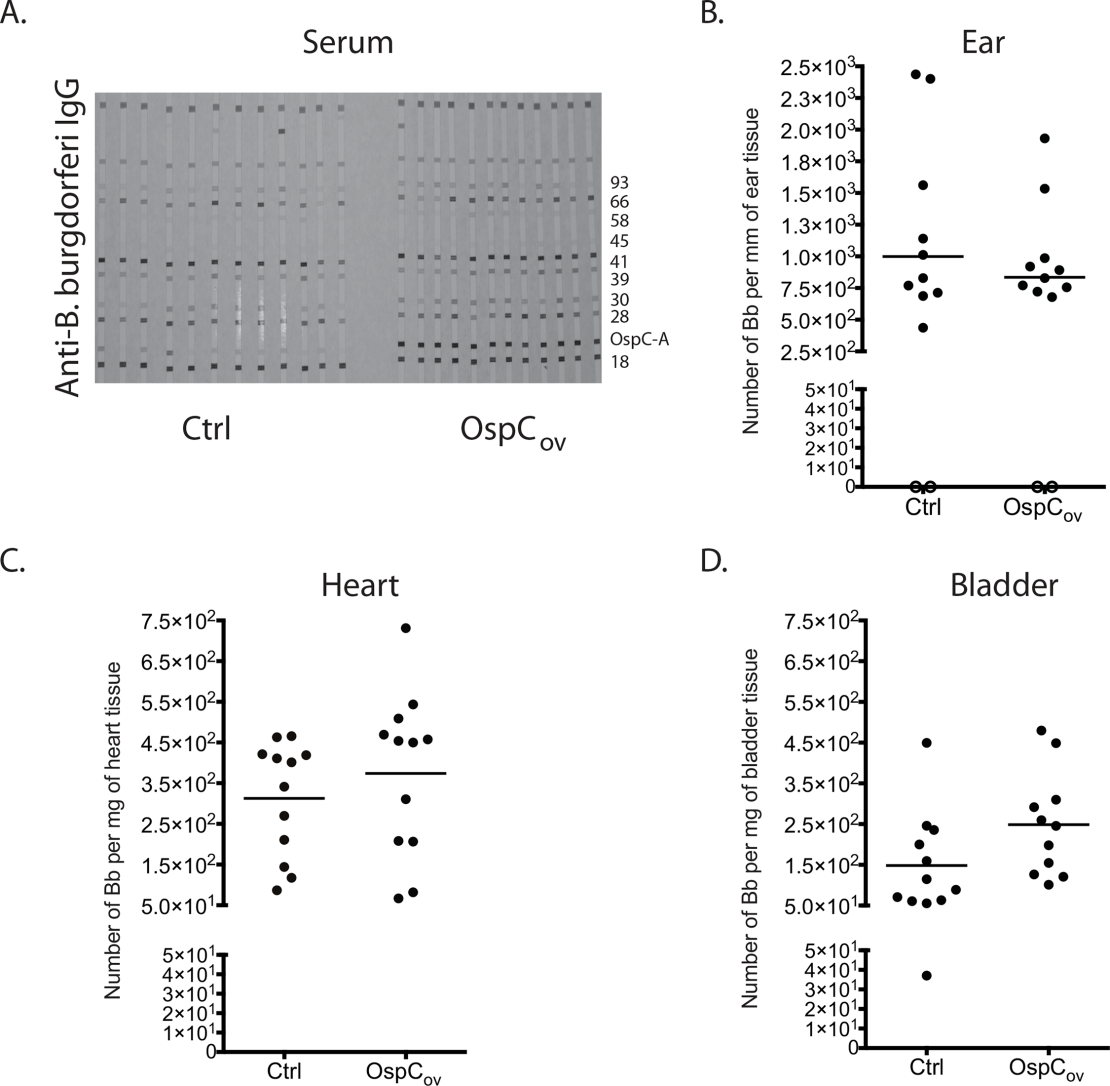
*B*. *burgdorferi* dissemination after tick challenge: serological evidence and spirochete burden in tissues. Mice (n = 12/group) were immunized intragastrically with *E*. *coli* expressing OspC-K or empty vector control (Ctrl) and then challenged with flat nymphs infected with *B*. *burgdorferi*. Serum samples were collected after challenge and IgG antibody response to *B*. *burgdorferi* analyzed by Western blot (3A). Each stripe represents one mouse and a pattern of at least 5 out of 10 positive bands are considered evidence of infection. Ear, heart and bladder samples were collected for DNA extraction followed by qPCR analysis (3B, 3C, 3D). qPCR data is expressed in number of *B*. *burgdorferi* per mm of ear tissue, and per mg of heart or bladder tissue.

We analyzed the levels of IgG to *B*. *burgdorferi* in serum from mice after nymphal challenge by Western blot. All vaccinated and unvaccinated mice seroconverted to *B*. *burgdorferi* (>5 positive bands); 100% of mice vaccinated orally with *E*. *coli* expressing OspC-K produced antibodies that bound with high avidity to OspC type A and 8/12 (67%) of unvaccinated mice developed antibodies that bound somewhat weakly to OspC type A in the Western blot (Fig 3A).

#### Quantification of *B*. *burgdorferi* burden in immunized mouse tissues

Spirochetal dissemination to target tissues such as ear, heart and bladder was determined by culture analysis via dark field microcopy. We also extracted DNA from these tissues and quantified the number of *B*. *burgdorferi* by q-PCR (Fig 3B, 3C and 3D). All mice immunized orally with OspC-K had positive cultures of *B*. *burgdorferi* in heart and bladder, confirmed by positive q-PCR with an average number of and 3.7 x 10^2^ *B*. *burgdorferi* per mg of heart (Fig 3C) and 2.5 x 10^2^ *B*. *burgdorferi* per mg of bladder tissue (Fig 3D). Furthermore, we detected positive cultures of *B*. *burgdorferi* from 10 ears out of 12 immunized mice with an average number of 8.3 x 10^2^ *B*. *burgdorferi* per mm of ear sample (Fig 3B). We found no significant differences in numbers of *B*. *burgdorferi* between control and tissues from mice orally immunized with *E*. *coli* expressing OspC-K, *p*>0.05.

#### Analysis of engorged nymphs

**Fig 4:**
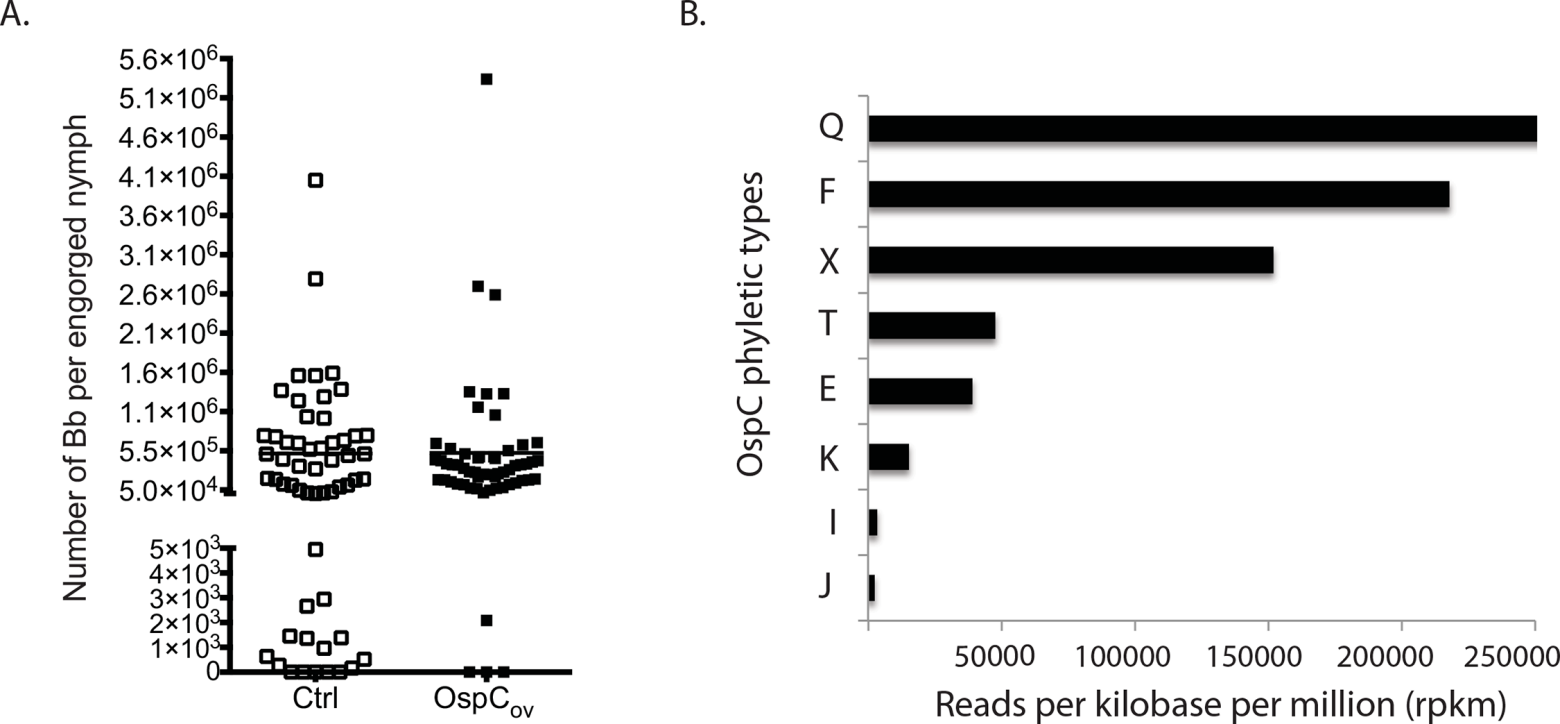
Analysis of engorged nymphs after challenge. Nymphs that fed on OspC immunized mice or on control mice became engorged and were collected for qPCR and next generation sequencing analysis. qPCR data (4A) is expressed in number of *B*. *burgdorferi* per tick. Sequence data (4B) was aligned by BOWTIE2 against 24 OspC reference strains using 1% mismatch stringency.

Engorged nymphs collected after challenge were subjected to DNA extraction followed by q-PCR targeting the FlaB gene (Fig 4A) and by next generation sequence analysis (Fig 4B). Nymphs (n = 115) that fed on immunized mice had an average number of 5.2 x 10^5^ *B*. *burgdorferi*, which was not different from the average number *B*. *burgdorferi* per nymph that fed on the control group (5.2 x 10^5^), *p*>0.05 (Fig 4A). Genomic DNA purified from 10 engorged nymphal ticks that fed on vaccinated mice was pooled and sent for next generation sequencing using Illumina MiSeq (Genewiz, South Plainfield, NJ) using general primers for OspC. Sequence data aligned against 24 published reference strains using 1% mismatch stringency identified OspC types Q, F, X, T, E, K, I, J (Fig 4B). A second alignment against 147 OspC reference sequences using 5% mismatch stringency identified three additional OspC types: A, D, M. Overall, we identified the following OspC phyletic types: A, D, E, F, I, J, K, M, Q, T, X in our laboratory infected ticks that fed on OspC-K vaccinated mice.

#### Analysis of *B*. *burgdorferi* OspC phyletic types in ticks and tissues

**Fig 5:**
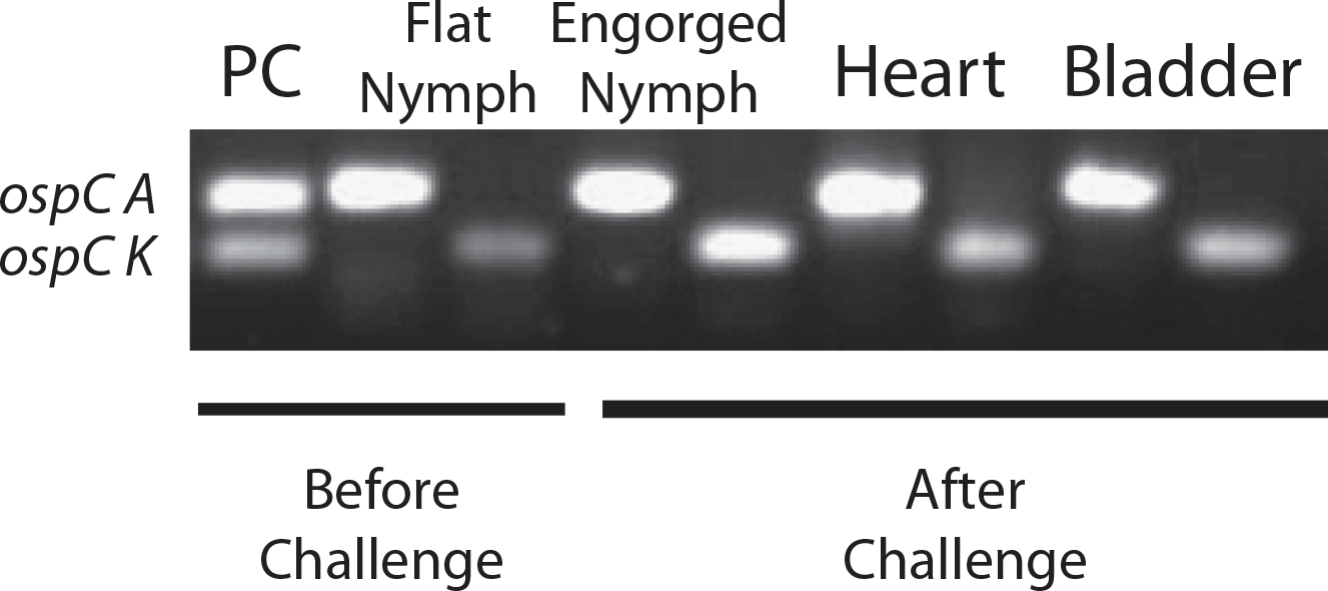
Analysis *B*. *burgdorferi* OspC phyletic types in flat nymphs, engorged nymphs and tissues. 1% agarose gel electrophoresis showing direct PCR products after gene amplification of DNA extracted from a culture containing multiple strains of *B*. *burgdorferi* (positive control, PC), flat and engorged nymphs and tissues (heart and bladder) with OspC type A primers (F: CCGAAAATAATCACAATGGATC; R: CCAAGTTCTTCAGCACC, 256bp) and OspC type K primers (F: GAAGCGGGGCATAATGGAA; R: CGCATGTTCTCCTTCTAGT, 165bp).

We analyzed OspC phyletic types from *B*. *burgdorferi* in cultures, flat and engorged nymphs and in tissues collected after challenge by direct PCR. We targeted our vaccine gene, *ospC* type K (fragment ~165bp) and *ospC* type A (fragment ~256bp) as an example of dissemination of other invasive OspC phyletic types (Fig 5). A culture containing multiple phyletic types of *B*. *burgdorferi* used to produce infected flat nymphs for challenge was tested as positive control. We amplified *ospC* type A and *ospC* type K from flat nymphs and from nymphs that engorged feeding on OspC-K immunized mice and from the heart and bladder of the same mice. There were no differences between vaccinated and unvaccinated groups.

### Assessment of vaccine failure

#### Differences in IgG isotypes in serum from orally immunized mice after tick challenge

**Fig 6:**
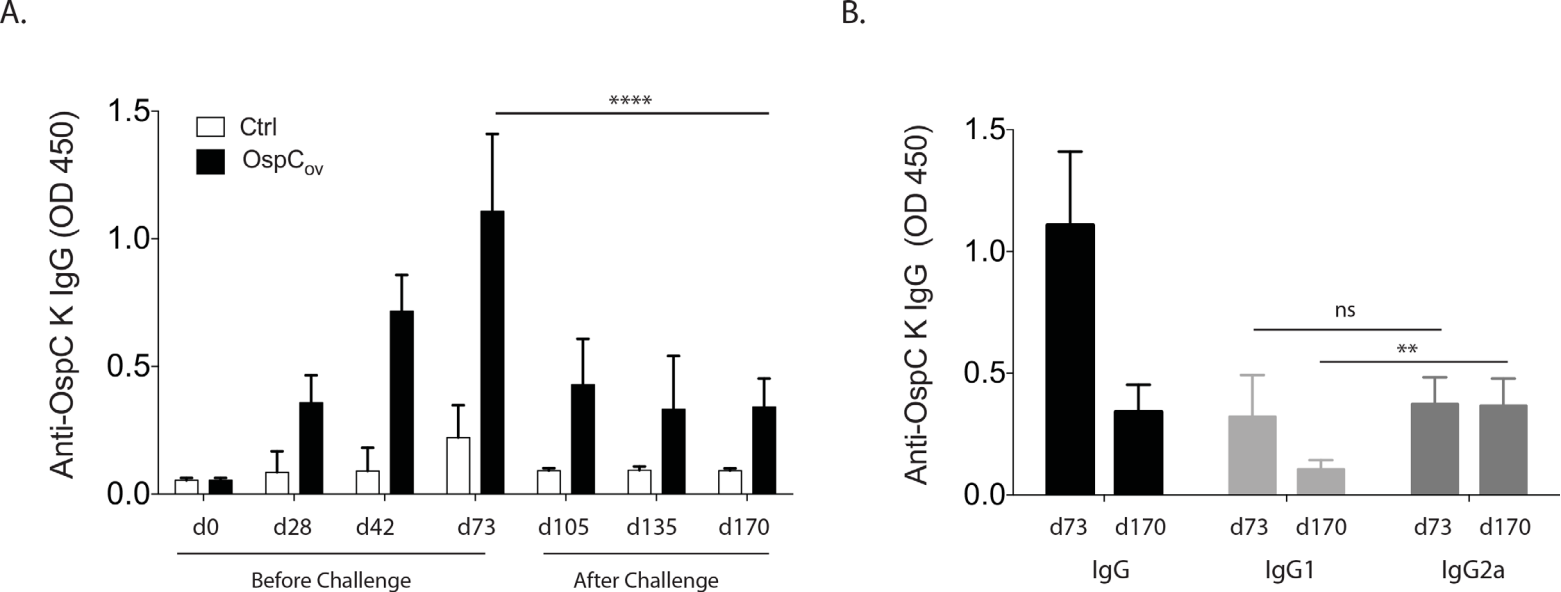
Differences in IgG isotypes in serum from orally immunized mice after tick challenge. Mice (n = 12) were immunized intragastrically and then challenged with flat nymphs infected with *B*. *burgdorferi* on day 73. Serum samples were collected before and after immunization (days 0, 28, 42 and 73) and after tick challenge (days 105, 135 and 170) and serum was analyzed for OspC specific IgG antibody by ELISA (6A). We analyzed differences between IgG isotypes on days 73 and 170 (6B). The results are plotted as Optical Density at 450 nm (OD450). The average of triplicate samples per mouse was determined and the error bar indicates standard deviation. Legend: ns, not significant; ** *p*<0.005, **** *p*<0.0001, unpaired T test with equal SD.

We compared the levels of OspC-K specific IgG antibodies in serum following oral immunization of mice before (days 28, 42 and 73) and after tick challenge (days 105, 135 and 170) by ELISA (Fig 6). IgG antibody to OspC-K significantly increased as a result of immunization on days 1–5/8–12 and boosting on days 29–33 and 43–47; on the day of tick challenge (day 73) it reached the highest level compared to control, *p*<0.0001. However, after challenge (between days 73 and 105) IgG levels to OspC-K dropped to levels below OD_450_<0.5 and remained low until mice were terminated on day 170, *p*<0.0001 (Fig 6A). We looked at differences between IgG isotypes between d73 and d170 to determine if the drop in total IgG after challenge was due to the loss of IgG1, IgG2a, or both (Fig 6B). Differences between IgG1 and IgG2a were not significant on the day of challenge, d73 (*p* = 0.3820), but we measured a significant loss in IgG1, not IgG2a, from d73 to day 170 (*p* = 0.0012), 3 months after challenge.

#### Anti-OspC borrelicidal activity

**Fig 7:**
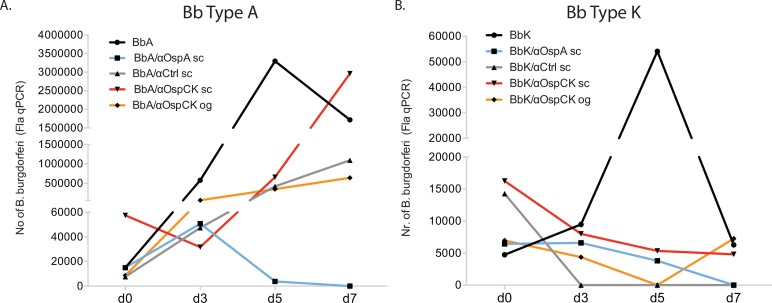
Assessment of heterologous and homologous OspC type K antibody borreliacidal activity. 10^4^ *B*. *burgdorferi* (Bb) type A and type K were cultured in the presence of serum (complement intact) from mice orally immunized with OspC-K (orange line) as well as mice immunized subcutaneously with OspC-K (red line), OspA (blue line) and Control (grey line); cultures were incubated at 34°C for 7 days. Samples were collected on d0, d3, d5 and d7 post-treatment for quantification of number of spirochetes by FlaB qPCR. Bb, *B*. *burgdorferi*; BbA, *B*. *burgdorferi* type A; BbK, *B*. *burgdorferi* type K; sc, subcutaneous immunization; og, oral gavage immunization.

Type A and type K *B*. *burgdorferi* (10^4^) were treated with serum from mice immunized orally with *E*. *coli* expressing OspC-K, and immunized subcutaneously with purified recombinant OspC-K, OspA and control; cultures were incubated at 34°C for 7 days. Samples were collected on days 0, 3, 5 and 7 post-treatment for visualization of spirochetes under a dark field microscope (DFM) and quantification by qPCR targeting the Fla gene (Fig 7). On day 7 post-treatment, both *B*. *burgdorferi* type A and type K survived and grew in culture (DFM) supplemented with intact serum from mice immunized with OspC-K orally or subcutaneously. We quantified ~10^5^ Type A *B*. *burgdorferi* in cultures treated with anti-OspC-K serum generated either by oral gavage or by subcutaneous injection (Fig 7A) and ~10^3^ Type K *B*. *burgdorferi* treated with anti-OspC-K serum generated either by oral gavage or by subcutaneous injection (Fig 7B). As a positive control for borrelicidal activity, we treated type A and type K spirochetes with serum from mice immunized with OspA, which killed both types of spirochetes. Overall, in a biological environment in which *B*. *burgdorferi* comes in contact with serum containing active complement both types of spirochetes survived in the presence of OspC-K serum whereas the spirochetes were killed by OspA serum.

## Discussion

We developed a series of oral vaccines against Lyme disease and found that only OspC induced significant amounts of antigen specific IgG and IgA antibodies after oral immunization. However, OspC did not protect mice from infection with heterologous or homologous types of *B*. *burgdorferi* when the spirochete was presented to the host in the natural context of transmission via challenge with *I*. *scapularis* infected with multiple strains of *B*. *burgdorferi*.

Analysis of localization and lipidation of the five vaccine constructs in the live bacterial carrier (*E*. *coli*) revealed that all immunogens were incorporated mostly within the cell envelope and that OspC, BBK32 and Salp 15 were most prevalent within the hydrophobic portion of the extract, CE/DET, (Figs 1, S1B and S1D) as we previously demonstrated for other oral vaccines candidates against Lyme disease [13], plague [14], and Leptospirosis [16]. Interestingly, OspC and Salp15 induced antigen specific IgG responses in blood from orally immunized mice but only OspC induced significant levels of IgA locally in the gut as well as in sites distal to the inoculation site, in the lungs and in the vagina (Fig 2 and S2 Fig). Our data showed that only mice immunized with *E*. *coli* expressing OspC induced significant IgG and IgA antibody responses suggesting broad involvement of systemic immunity after oral immunization. Thus, vaccine efficacy was evaluated following oral immunization with *E*. *coli* expressing OspC-K.

We used two tests to assess vaccine efficacy after challenge: seroconversion to *B*. *burgdorferi* and quantification of *B*. *burgdorferi* DNA in tissues (Fig 3). Analysis of serum from C3H-HeN mice tested against a commercial Western blot assay commonly used for diagnosis of human Lyme disease showed no difference between seroprofiles of vaccinated and unvaccinated controls other than the expected solid band for OspC in vaccinated mice. 67% of unvaccinated mice developed antibodies to OspC (Fig 3A) as reported by other investigators for naturally infected *P*. *leucopus* [31]. We also cultured *B*. *burgdorferi* from all tissues and quantified about 250 to 1000 spirochetes in ear, heart and bladder tissues of vaccinated and unvaccinated controls (Fig 3B, 3C and 3D) which represents about 0,1% of the total number of spirochetes present in engorged ticks (Fig 4A). Our data demonstrates that oral immunization with *E*. *coli* expressing OspC type K did not prevent infection with *B*. *burgdorferi* after tick challenge.

The ticks used for challenge were produced from a culture of *B*. *burgdorferi* isolated from heart of *P*. *leucopus* infected with field caught ticks from an endemic area of Lyme disease. Given that there are at least 25 phyletic types of OspC [32], [33], [34] and that seven (A, B, C, D, I, K, N) cause disseminated infection in humans, [35], [36] we investigated the types of OspC present in ticks used for challenge (Fig 4B). Next generation sequencing analysis of genomic DNA purified from engorged nymphal ticks identified 11 OspC phyletic types: A, D, E, F, I, J, K, M, Q, T, X, four of which were invasive types A, D, I, K. We confirmed by direct PCR that these ticks contained *B*. *burgdorferi* type K in addition to type A, which we used as an example of another invasive OspC type (Fig 5). Furthermore, we show the presence of *B*. *burgdorferi* containing the same pair of OspC phyletic types in heart and bladder from mice orally immunized with OspC-K showing that oral immunization did not prevent dissemination of *B*. *burgdorferi* carrying the homologous type of OspC (type K) nor the heterologous type A. Others have shown that OspC protection is type specific after challenge with homologous strains via needle challenge [30], [37] and also via tick challenge [22], but not after challenge with heterologous strains via needle challenge [37].

To understand some of the mechanisms that lead to vaccine failure we evaluated the OspC-specific antibody response during immunization and after challenge (Fig 6) and we performed a borreliacidal assay using serum from immunized mice collected the day before challenge (Fig 7).

Seven weeks after the last immunization and four weeks after tick challenge (day 105), antibody production to OspC decreased to levels below OD_450_ <0.5, (Fig 6A) which is the minimum necessary to ensure some level of protection against OspA [7]. Levels of antibodies remained low until day 170, three months after challenge. These data suggest that production of antibody to OspC by the host depends on the presence of OspC pressure via immunization as we previously observed for OspA [5]. In a study to investigate the anamnestic immune response to *B*. *burgdorferi*, Gilmore et all found that immunization with OspC type A waned quickly and did not protect from challenge with naturally infected ticks [38]. Alternatively, production of anti-OspC antibody may have been down regulated by the tick inoculation of *B*. *burgdorferi* during feeding. Furthermore, *B*. *burgdorferi* is able to negatively regulate OspC expression in presence of anti-OspC antibodies as a mechanism of immune evasion [39], [40], [41]. If OspC is not presented on the surface of the spirochete then antibodies against OspC will not find the protein for recognition and additional immune response processing and killing. The fact that synthesis of OspC by *B*. *burgdorferi* is involved exclusively in transmission from tick to mammal and not from mammal to tick [42] lends further support to our hypothesis.

In this study we also show that oral vaccination of mice with OspC induced significant levels of antigen-specific IgG equally distributed between IgG1 and IgG2a before we infected mice via tick challenge. After tick challenge, total IgG was reduced to about half and we observed that the difference was due to the loss of IgG1, an immunoglobulin subclass associated with TH2 responses, but not IgG2a an immunoglobulin associated with TH1 responses (Fig 6B). Interestingly a study by Caine et al shows that elimination of OspC decreases bloodstream burdens of *B*. *burgdorferi* in TH1 biased C3H-HeN but not in TH2 biased Balb/c mice [43] suggesting that OspC expression is necessary for *B*. *burgdorferi* dissemination in the TH1 C3H-HeN background but not in TH2 Balb/c. In our study we observed that in the absence of *B*. *burgdorferi* expressing OspC, the mouse (C3H-HeN) produced equal levels of IgG1 and IgG2a to the OspC immunogen indicative of a TH1/TH2 response before challenge. After the host was exposed to *B*. *burgdorferi* expressing OspC upon challenge, dissemination of *B*. *burgdorferi* proceeded efficiently in the presence of anti-OspC antibody and the immune response seems to have switched from the initial TH1/TH2 response raised against the OspC-K immunogen, to a TH1 biased response to the pathogen.

Finally, our borrelicidal assay data suggested that challenge with *B*. *burgdorferi* carrying an OspC distinct from (type A) or homologous to (type K) the vaccinogen (type K) may have lead to immune evasion given that in either case we were able to quantify increasing amounts of live type A and type K spirochetes after treatment with serum from OspC-K immunized mice which was independent of the route of immunization (Fig 7). Our data seems to corroborate previous findings in that pathogenic *B*. *burgdorferi* impairs the complement pathway of the host and thus survives in serum [44]. The results we observed for anti-OspC borrelicidal activity support Bockenstedt’s findings in that neutralization activity was weak even against autologous strains [30]. Although we observed a degree of type-specific killing with anti-OspC type K mouse serum (loss of 1log of spirochetes after treatment) it was nevertheless insufficient. Others have shown that anti-OspC borrelicidal activity is dependent on complement and that serum produced against a cocktail of OspC types (α-ABKD) killed each of the 4 types of spirochetes included in the vaccine cocktail [24]. In that study, the authors did not investigate killing of each type of spirochete by its respective type-specific serum. Thus, it is possible that the OspC strain cross-protection observed may be related to the effect of one type of OspC in the cocktail rather than all four, and it certainly does not mean that serum containing all types of OspC antibodies will kill all types of *B*. *burgdorferi*.

Our results suggest that oral vaccines based in OspC type K are ineffective. Vaccine failure in mice could be due to factors related to the biology of the spirochete (*B*. *burgdorferi* early shut down of OspC expression on its surface), due to factors related to the immunological response to the vaccine (ie, the quick waning of the antibody response to OspC after immunization or due to tick induced shutdown of B cell production of IgG antibodies), due to the genetic background of the mice (OspC expression is needed for *B*. *burgdorferi* dissemination in C3H-HeN but not in Balb/c mice), and to partial neutralization of *B*. *burgdorferi* by homologous serum and inefficient production of cross-protective neutralizing borreliacidal antibodies to OspC by the mammalian host as shown here and in [25], [23].

## Supporting Information

S1 Fig Localization of recombinant OspB, BBK32, Salp25 and Salp15 in *E*. *coli*. Cells expressing recombinant proteins were disrupted with a French press and supernatant (SN), cell envelope (CE), and cell envelope partitioned into detergent (CE/DET) and aqueous (CE/AQ) phases were analyzed by cell fractionation followed by quantification of protein by densitometry using an Alpha Imager (Alpha Innotech, San Leandro, CA).
(EPS)

S2 Fig Serological IgG and mucosal IgA response in mice immunized orally with recombinant *E*. *coli* expressing *B*. *burgdorferi* genes *ospB*, *bbk32* and *Ix scapularis* genes *salp25* and *salp15* on the day before challenge. C3H/HeN mice were vaccinated orally with live recombinant *E*. *coli* expressing OspB, BBK32, Salp25, Salp15; control mice were orally inoculated with *E*. *coli* carrying the empty vector (C). C3H/OspB and BBK32, n = 6 per group; C3H/Salp25 and Salp15 n = 4 per group. Blood, stool (GUT), bronchoalveolar lavage (BAL) and vaginal lavage (VAL) samples were collected on the day before challenge and antigen-specific IgG, subtyped into IgG1 and IgG2a (S2A Fig) and antigen-specific IgA (S2B Fig) were measured by ELISA and plotted as Optical Density at 450 nm (OD 450). The average of triplicate readings per mouse/per group was determined and the error bar indicates standard deviation.
(EPS)
